# Nanoparticles for Improved Local Retention after Intra-Articular Injection into the Knee Joint

**DOI:** 10.1007/s11095-012-0870-x

**Published:** 2012-09-21

**Authors:** Michael Morgen, David Tung, Britton Boras, Warren Miller, Anne-Marie Malfait, Micky Tortorella

**Affiliations:** 1 Bend Research Inc., 64550 Research Road, Bend, Oregon 97701 USA; 2 BioMed Valley Discoveries, Kansas City, Missouri USA; 3 Departments of Medicine and Bioengineering, University of California—San Diego, San Diego, California USA; 4 Rush University Medical Center, Chicago, Illinois USA; 5 Guangzhou Institute of Biomedicine and Health, Chinese Academy of Sciences, Beijing, China

**Keywords:** cross-linked hydrogels, increased retention, intra-articular injection, nanoparticles

## Abstract

**Purpose:**

To evaluate using cationic polymeric nanoparticles that interact with hyaluronate to form ionically cross-linked hydrogels to increase the intra-articular retention time of osteoarthritis drugs in the synovial cavity.

**Methods:**

*In vitro* tests included nanoparticle release from cross-linked hydrogels using syringe and membrane dissolution tests, viscosity measurement of synovial fluid containing hydrogels, and release-rate measurement for a model active conjugated to a cationically substituted dextran using a hydrolyzable ester linkage in a sink dissolution test. Nanoparticle retention after intra-articular injection into rat knees was measured *in vivo* using fluorescence molecular tomography.

**Results:**

Diffusional and convective transport of cationic nanoparticles from ionically cross-linked hydrogels formed in synovial fluid was slower *in vitro* than for uncharged nanoparticles. Hydrogels formed after the nanoparticles were mixed with synovial fluid did not appreciably alter the viscosity of the synovial fluid *in vitro*. *In vitro* release of a conjugated peptide from the cationic nanoparticles was approximately 20% per week. After intra-articular injection in rat knees, 70% of the nanoparticles were retained in the joint for 1 week.

**Conclusions:**

This study demonstrates the feasibility of using cationic polymeric nanoparticles to increase the retention of therapeutic agents in articular joints for indications such as osteoarthritis.

## Introduction

With an aging U.S. population, the prevalence of arthritis is expected to increase in the coming decades. By the year 2030, an estimated 67 million adults aged 18 years and older (25% of the projected total adult population) will have doctor-diagnosed arthritis, compared with the approximately 43 million diagnosed adults in 2002. Two-thirds of those with arthritis will be women. By 2030, an estimated 25 million adults (9.3%) will report arthritis-attributable activity limitations. These estimates may be conservative, because they do not account for the current trends in obesity, which may contribute to future cases of osteoarthritis (OA) (1).

Currently, medical management of OA, the most prevalent form of arthritis, focuses on control of symptoms, particularly of pain. Management of mild to moderate OA pain can be accomplished using nonsteroidal anti-inflammatory drugs (NSAIDs), with COX-2 inhibitors taking a significant market share from older members of this class.

Several new classes of molecules have been discovered that inhibit one or more OA pathophysiological processes. Recent preclinical studies have demonstrated that these potentially disease-modifying osteoarthritis drugs (DMOADs) can block specific key disease mechanisms and retard the progression of structural changes in animal models of OA (2,3). While promising targets for the development of DMOADs have been identified, therapeutic agents that target these proteins are often not suitable for systemic administration, frequently due to toxicity associated with the target protein, especially in the context of chronic treatment.

Because the goal is to modulate these targets primarily in the affected joint, intra-articular administration of compounds is an attractive treatment modality. Intra-articular administration of a DMOAD directly into the affected joint offers several advantages over oral dosing; for example, it can be used for compounds that are poorly absorbed, have undesirable systemic pharmacokinetic (PK) profiles, or have systemic toxicity.

Since most agents are rapidly cleared from the joint after intra-articular injection, the biggest challenge in the development of intra–articular DMOADs is to increase retention time of the therapeutic agent in the joint. For example, the half life of NSAIDs has been reported to be as short as 1 to 5 h after local injection (4). This short duration of action requires frequent injections, which in turn results in expensive treatment, poor patient compliance, and injection-associated complications (e.g., infection, post-injection flare, crystal-induced synovitis, cutaneous atrophy) (5).

Many types of particulate carriers have been investigated for increasing the retention time of therapeutic agents within the knee cavity, including the following:

- liposomes (6–11);
- microparticles of poly(lactide) (PLA), poly-L-lactide acid (PLLA), and poly(lactide-co-glycolide) (PLGA), including hyaluronate-functionalized PLA/PLGA (12–18);
- albumin microparticles (19,20);
- chitosan (21);
- magnetic nanoparticles (22,23);
- solid lipid nanoparticles (24);
- thermally responsive elastin-like polypeptides (ELPs) (25);
- pH-sensitive gels (26); and
- nanoparticles targeted to the cartilage using phage display peptides (27).

Some investigators have reported lower systemic exposure with such approaches. For example, Liang *et al.* showed that PLLA microspheres decreased systemic exposure 7- to 10-fold compared with free drug after intra-articular injection in the knees of rabbits (28). However, the intra-articular retention times for therapeutic agents with these approaches is still too short for maximum therapeutic effect or the approaches are subject to other limitations (e.g., they are applicable only to a narrow range of active compounds or involve the use of materials with unverified safety profiles). Thus, although a number of approaches have been studied, the challenge remains for achieving prolonged retention with a platform that is amenable to local delivery to the joint, has high drug loading capacity, and has a good safety profile.

**Fig. 1:**
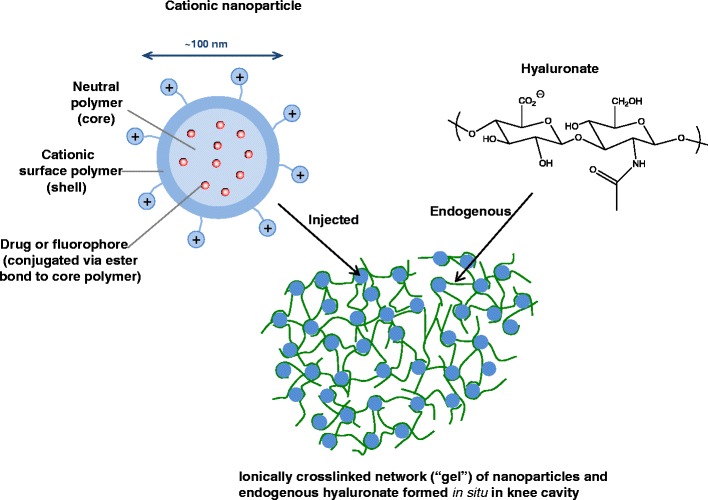
Nanoparticle architecture and mechanism of retention.

In this paper, we report the use of cationic polymeric nanoparticles that form diffuse ionically associated filamentous structures (“ionically cross-linked hydrogels”) with resident hyaluronate in the synovial cavity after intra-articular injection (Fig. 1). These nanoparticles were shown to increase the retention time in the knee of a small fluorescent peptide cargo (as a drug mimic) that was covalently bound to the polymeric nanoparticle via a hydrolyzable ester linkage.

## Materials and Methods

### Materials

Ethyl cellulose (Ethocel® Viscosity 4) was a generous gift from Dow Chemical Co. (Midland, MI). Eudragit RL100 was a generous gift from Evonik Industries (Essen, Germany). Poly[2-methoxy-b-(2-ethylhexyloxy)-1,4-phenylenevinylene] (MEH-PPV) (Product No. 541435) was purchased from Sigma Aldrich Corp. (St. Louis, MO). Polycaprolactone-b-co-polyethyleneoxide (PCL-PEO, Product No. P3128EOCL, 10-kDa PCL, 5-kDa PEO) was purchased from Polymer Source Inc. (Montreal, Quebec, Canada). VivoTag® 680 (Product No. 10120) was purchased from VisEn Medical Inc. (Woburn, MA). The lyophilized potassium salt of hyaluronic acid (“hyaluronate,” Product No. 53730) from human umbilical cord was purchased from Fluka (St. Louis, MO). Human synovial fluid from OA patients (Part No. HYSYNOV-OA) was purchased from Bioreclamation Inc. (Hicksville, NY). A tetrapeptide labeled with fluorescein isothiocyanate (FITC) was provided by Pfizer Inc. (St. Louis, MO). RFK peptide labeled with fluorescein isothiocyanate (RFK-FITC) was purchased from American Peptide Company, Inc. (Sunnyvale, CA). Phosphate buffered saline (PBS “10X,” Product No. AM9624) for *in vitro* hydrolysis testing was obtained from Invitrogen/Life Technologies (Carlsbad, CA).

Syringe filters (1-μm glass-microfiber membrane and 0.2-μm polyethersulfone [PES] Supor filters) were purchased from Pall Corp. (Port Washington, NY). Methylene chloride (Product No. BDH1113) was purchased from VWR International LLC (Radnor, PA). Porous polypropylene membranes (Accurel® PP 1E R/P) were purchased from Membrana GmbH (Wuppertal, Germany).

The detailed synthesis of the following dextran derivatives used in the studies, made by derivatization of either 10-kDa dextran (Dextran 10) or 20-kDa dextran (Dextran 20), will be described in a separate publication:

- Dextran 10 propionate succinate (D10PS)-RFK-FITC;
- Dextran 20 acetate quaternary amine-Texas Red (D20AQA-TR);
- Dextran 20 propionate (D20P)-VivoTag; and
- Dextran 10 propionate (D10P).

**Fig. 2:**
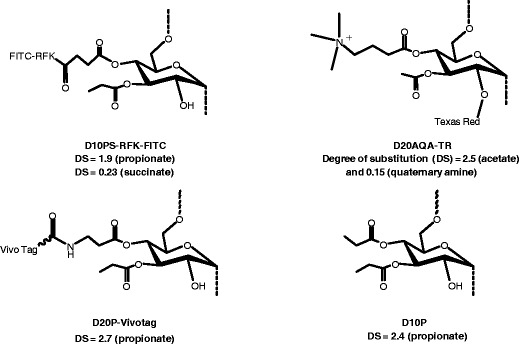
Structures of dextran derivatives. DS is the degree of substitution and refers to the average number of hydroxyls per saccharide monomer substituted.

The structures of these polymeric derivatives are shown in Fig. 2.

### Methods

#### Nanoparticle Manufacture

The nanoparticles are comprised of two polymers: a neutral (i.e., uncharged) core polymer and a cationic surface polymer that interacts with the resident hyaluronate in the joint to form ionically associated or “cross-linked” structures (Fig. 1).

A number of nanoparticle formulations were manufactured to demonstrate the *in vitro* and *in vivo* capabilities of the platform. Eudragit RL100 at 20- to 25-wt.% loading was used as a model cationic surface polymer for all studies, with the exception of one *in vivo* imaging test in which a cationic dextran was used to show the flexibility in choice of a surface polymer to associate with hyaluronate. The neutral polymer, which was expected to have less influence on the nanoparticle interaction with hyaluronate, was varied for different tests for convenience or for optimal incorporation of model actives. For *in vitro* imaging tests, formulations incorporating a polymeric fluorescent dye were used to visualize the ionically cross-linked structures that were formed. Nanoparticles made with both Eudragit RL100 and cationically substituted dextran (29) were imaged after mixing with human OA synovial fluid to demonstrate the flexibility of using various chemistries to form ionically cross-linked structures. Uncharged nanoparticles made with PCL-PEO were used as controls to compare with the cationic nanoparticles. For the *in vitro* hydrolysis experiments and the *in vivo* test, a small-molecule dye was covalently conjugated via an ester group to a hydrophobically substituted dextran “core” polymer to mimic the likely performance of an ester-linked drug molecule. The nanoparticle compositions are summarized in Table I and described below.

**Table I:** Summary of Nanoparticle Compositions and Corresponding Tests

Nanoparticle composition	Test
D20AQA-TR	Fluorescence imaging
75:20:5 D10P:Eudragit RL100:MEH-PPV	Fluorescence imaging
70:25:5 ethyl cellulose:Eudragit RL100:MEH-PPV	Viscosity, membrane dissolution
71:24:5 D10P:Eudragit RL100:MEH-PPV	Syringe-filter dissolution
74:21:5 ethyl cellulose:PCL-PEO:MEH-PPV	Syringe-filter dissolution (control)
74:25: 1 ethyl cellulose:PCL-PEO:MEH-PPV	Membrane dissolution (control)
D10PS-RFK-FITC	Hydrolytic release
37.5:37.5:25 D10P:D20P-VivoTag:Eudragit RL100	*In vivo* retention

D20AQA-TR nanoparticles were prepared for fluorescence imaging by dissolving 30 mg of D20QA-TR in 0.5 ml of methanol (MeOH) by vortexing for 15 min. This solution was injected into 5 ml of water over the course of approximately 1 s, stirred at 60 rpm, and then rotoevaporated to remove the MeOH.

75:20:5 D10P:Eudragit RL100:MEH-PPV nanoparticles were prepared for fluorescent imaging by dissolving 37.5 mg of D10P and 12.5 mg of Eudragit RL100 in 5 ml of tetrahydrofuran (THF) in which 2.5 mg of MEH-PPV was dissolved. This solution was injected into 50 ml of water. The solution was rotoevaporated to remove THF, leaving 8 ml of suspension. The suspension was then filtered through a 1-μm syringe filter before use.

70:25:5 ethyl cellulose:Eudragit RL100:MEH-PPV nanoparticles were prepared for the viscosity and membrane dissolution tests by dissolving 350 mg of ethyl cellulose, 125 mg of Eudragit RL100, and 25 mg of MEH-PPV in 5 ml of methylene chloride. This solution was then mixed with 20 ml of Milli-Q water using a rotor stator (Polytron 3100, Kinematica Inc., Bohemia, NY) at 10,000 rpm for 3 min. This coarse emulsion was further emulsified at 12,500 psi for 6 min using a Microfluidizer M110S (Microfluidics, Newton, MA) fitted with a Z-shaped interaction chamber with a 100-μm-diameter channel. The emulsion was then placed on a rotoevaporator, where the methylene chloride was removed under reduced pressure at approximately 25°C. The resulting aqueous suspension was filtered through a 1-μm glass-microfiber syringe filter.

Methods similar to those used for the 75:20:5 D10P:Eudragit RL100:MEH-PPV nanoparticles were used to prepare the 74:21:5 ethyl cellulose:PCL-PEO:MEH-PPV and 71:24:5 D10P:Eudragit:MEH-PPV nanoparticles for the syringe-filter dissolution test and the 74:25:1 ethyl cellulose:PCL-PEO:MEH-PPV nanoparticles for the membrane dissolution test.

D10PS-RFK-FITC nanoparticles were prepared for hydrolytic release studies using methods similar to those used for the D20AQA-TR nanoparticles. 37.5:37.5:25 D20P-VivoTag:D10P:Eudragit RL100 nanoparticles were prepared by dissolving 22.5 mg of D20P-VivoTag in 1.2 ml of methylene chloride and then passing the solution through a 0.2-μm filter. Eudragit RL100 (15 mg) and D10P (22.5 mg) were then dissolved in this solution. Manipulation of the methylene chloride solution in open vessels was limited in order to minimize evaporation of the solvent. Water (6.2 ml) was added to this solution and nanoparticles were made by emulsification and filtered as described above.

Nanoparticle size was measured by dynamic light scattering (DLS) using a BI-200SM size analyzer with a BI-9000AT correlator (Brookhaven Instruments Corp., Long Island, NY). Nanoparticle size is reported as the effective hydrodynamic diameter determined using the cumulant cubic algorithm.

#### Fluorescence Microscopy

Fluorescence microscopy was performed to visualize the cross-linked hydrogel formed by cationic nanoparticles and human OA synovial fluid. D20AQA-TR and 75:20:5 D10P:Eudragit RL100:MEH-PPV nanoparticle suspensions (0.2 ml of 3.5 mg/ml in ‘1X’ PBS, pH 7.4), were added separately to 0.4 ml of human OA synovial fluid, gently mixed, then spread on glass slides and imaged by fluorescence microscopy (Nikon Eclipse E600 optical microscope, equipped with an ultraviolet–visible [UV/vis] lamp and Nikon epifluorescence filter block set).

#### Viscosity Measurement (With and Without Gel)

A cone-and-plate rheometer (TA Instruments AR1000, New Castle, DE) was calibrated with mineral oil (Viscosity N75, Catalog No. 9727-C41) (Cannon Instrument Company, State College, PA) and water. Measurements were made using a 6-cm-diameter steel flat disk with a gap of 200 μm at 37°C. For these tests, 1 ml of human OA synovial fluid was mixed with 0.25 ml water and placed on the disk. Shear stress was measured using a shear rate from 0.1 to 100 Pa. The test was repeated by mixing 1 ml of human OA synovial fluid with 0.25 ml of a 21.5-mg/ml solution containing 70:25:5 ethyl cellulose:Eudragit RL100:MEH-PPV nanoparticles.

#### *In Vitro* Syringe-Filter Dissolution Test for Convective Transport (Charged Versus Neutral Nanoparticles)

The ability of convective fluid transport to dislodge nanoparticles from the gel formed between the nanoparticles and the hyaluronate in synovial fluid was assessed *in vitro* using a syringe-filter dissolution test. Two types of nanoparticles were tested: 74:21:5 ethyl cellulose:PCL-PEO:MEH-PPV nanoparticles and 71:24:5 D10P:Eudragit RL100:MEH-PPV nanoparticles. For these tests, 0.33 ml of a 5-mg/ml nanoparticle suspension was mixed with 0.66 ml of human OA synovial fluid inside a glass syringe fitted with a 1-μm glass-fiber filter prefilled with 1 ml of human OA synovial fluid. A syringe pump was used to deliver the mixture at 0.04 ml/cm^2^/h through the filter. Fractions (0.2 ml each) were collected every hour for 7 h. Air was introduced into the syringe to facilitate initial mixing upon inversion of the syringe and to aid in expelling the fluid residing in the filter for the last few timepoints, when the syringe itself was empty. Nanoparticle transport through the filter was measured by fluorescence using a Spectramax M5e plate reader (Molecular Devices, Sunnyvale, CA). An excitation wavelength of 480 nm was used. Emissions were collected from 510 to 670 nm in 10-nm increments.

#### *In Vitro* Membrane Dissolution Test for Diffusive Transport (Eudragit Versus PCL-PEO)

The diffusive transport of nanoparticles from human OA synovial fluid through a semipermeable membrane was measured in an attempt to assess the potential for transport across the semipermeable synovial lining of the knee. This test employed a custom membrane apparatus that has been previously described (30). Briefly, in this apparatus, two compartments were separated by a polypropylene membrane with a pore size of 1 μm. On the donor side of the membrane apparatus, 2 ml of a 20-mg/ml nanoparticle suspension was combined with 4 ml of human OA synovial fluid. The cell was gently shaken briefly to mix. Water (6 ml) was placed in the receptor side of the apparatus. The apparatus was placed in a temperature-controlled box at 37°C. Both the donor and receptor media were stirred at 60 rpm. After 1 h, the cells were placed in sealed jars to eliminate evaporation. Aliquots of the receptor solution (230 μl each) were taken at several timepoints and the nanoparticle concentration was measured by fluorescence emission using a plate reader. An excitation wavelength of 480 nm was used. The fluorescence spectrum of the receptor solution was collected from 530 to 630 nm, and fluorescence at 590 nm was plotted. The test was performed for two types of nanoparticles: 70:25:5 ethyl cellulose:Eudragit RL100:MEH-PPV and 74:25:1 ethyl cellulose:PCL-PEO:MEH-PPV nanoparticles.

#### *In Vitro* Release Test of Fluorescently Tagged Peptide

A solution containing approximately 1 mg/ml of D10PS-RFK-FITC nanoparticles in PBS (Invitrogen “5X,” [i.e., “10X” diluted by a factor of 2]) was placed in an amber glass vial covered with aluminum foil inside a temperature-controlled box at 37°C and stirred at 60 rpm. Periodically, 100 μl aliquots were removed and mixed with 100 μl of MeOH. The RFK-FITC concentration of the sample (i.e., the concentration of the fluorescent tag) was analyzed using a Hewlett Packard Series 1100 high-performance liquid chromatography (HPLC) instrument (HP Development Corp., Palo Alto, CA) with a Phenomenex C18 column (300 Å, 250 mm by 4.6 mm). The following conditions were used: injection volume of 20 μl, column temperature of 20°C, and flow rate of 1.2 ml/min, with ultraviolet (UV) detection at 440 nm. A gradient method was used with the following ratios of Mobile Phase A to Mobile Phase B: 80:20, 48:52, and 0:100 at 0, 24, and 24.5 min, respectively, where Mobile Phase A was 0.1% tetrafluoroacetic acid (TFA) in water and Mobile Phase B was 0.1% TFA in acetonitrile (ACN).

The concentration of RFK-FITC released was determined by comparing results to that of a control solution with 100% hydrolysis, achieved by adding 1 ml of the 1-mg/ml nanoparticle suspension to 15 ml of 1 M NaOH (pH 11.5) and stirring overnight.

#### *In Vivo* Fluorescence Molecular Tomography (FMT)

The *in vivo* retention profile of the nanoparticle suspension was examined in the knee joints of 350-g female Sprague-Dawley® rats (Charles River Laboratories International Inc., Wilmington, MA). The animals were anesthetized with 3% isoflurane gas delivered by a laboratory animal anesthesia system (LAAS) (BioNimbus Inc., Fort Collins, CO). The knee region was shaved and disinfected with iodine solution and a 90% ethanol scrub. In the control group, 20 μL of VivoTag-labeled peptide (10 mg/ml) was injected into the right knee. In two separate test groups of animals, 10 μL of 10 mg/ml 37.5:37.5:25 D20P-VivoTag:D10P:Eudragit RL100 linked peptide was injected intra-articularly, followed by injection of 10 μL of either PBS or 2 mg/ml hyaluronic acid (*n* = 4 animals/group). The purpose of this dual-injection protocol was to assess whether the addition of extra hyaluronate (in addition to the endogenous level in the knee) would result in additional cross-linking and increased retention time. At designated timepoints up to 1 week, two animals from each group were sacrificed, and the intact knees were harvested. The skin of the knees was removed, and the samples were scanned with a VisEn FMT system to measure the fluorescence level (VisEn Medical Inc., Woburn, MA). All animal experiments were approved by the Pfizer Institutional Animal Care and Use Committee (IACUC) and met all regulatory guidelines.

## Results

### Nanoparticle Particle-Size Distribution

**Fig. 3:**
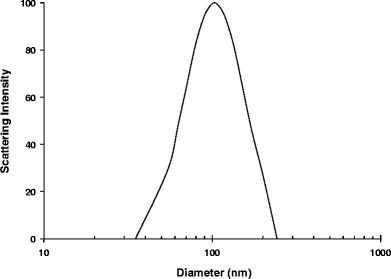
Particle-size distribution for 37.5:37.5:25 D10P:D20P-VivoTag:Eudragit RL100 nanoparticles that were dosed for *in vivo* retention studies. Scattering intensity frequency is plotted, as determined by a CONTIN fitting algorithm.

Nanoparticle size was determined using DLS. Effective diameters of all nanoparticles, as determined by cubic cumulant fits, were between 100 nm and 150 nm, with polydispersities ranging from 0.1 to 0.4. Figure 3 shows the particle-size distribution for the 37.5:37.5:25 D10P:D20P-VivoTag:Eudragit RL100 nanoparticle suspension used in the *in vivo* retention study. The zeta potential of the nanoparticles described here was not measured, but the authors have previously confirmed the expected positive zeta potential of similar cationic nanoparticles (data not shown).

### Fluorescence Microscopy

**Fig. 4:**
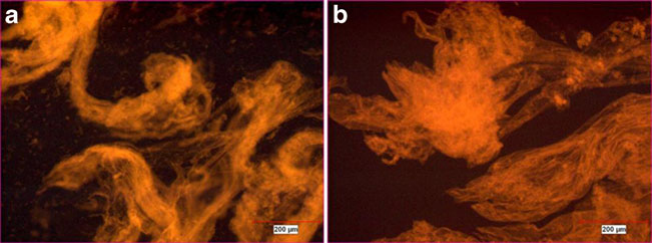
Fluorescent microscopic images of gelled 75:20:5 D10P:Eudragit RL100:MEH-PPV nanoparticles (**a**) and D20AQA-TR (i.e., dextran-based) nanoparticles (**b**) with human OA synovial fluid.

Fluorescence microscopy was used to visualize the morphology of the *in situ* gels formed after mixing the fluorescently labeled cationic nanoparticles with human OA synovial fluid. Figure 4a and b show optical micrographs of gels produced by mixing 75:20:5 D10P:Eudragit RL100:MEH-PPV nanoparticles and D20AQA-TR (i.e., dextran-based) nanoparticles with human OA synovial fluid.

Immediately upon mixing, a discontinuous, filamentous fluorescent gel formed in the human OA synovial fluid for both types of cationic nanoparticles. Similar structures formed when the same nanoparticles were mixed with solutions of hyaluronate (data not shown).

### Viscosity of Human OA Synovial Fluid

The effect of cationic nanoparticles on the viscosity of human synovial fluid was measured as a function of shear rate to determine if *in situ* formation of the hydrogel filaments altered the viscoelastic properties of the synovial fluid. This is an important consideration, because significant alteration of the rheological properties of the synovial fluid could adversely affect the function of the joint.

**Fig. 5:**
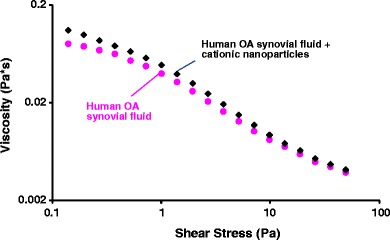
Viscosity as a function of shear stress of human OA synovial fluid alone and treated with cationic 75:20:5 D10P:Eudragit RL100:MEH-PPV nanoparticles.

Figure 5 shows dynamic viscosity as a function of shear stress at 37°C for human OA synovial fluid alone and treated with cationic 70:25:5: ethyl cellulose:Eudragit RL100:MEH-PPV nanoparticles. The formation of a gel with the cationic Eudragit-based nanoparticles increased the measured viscosity of the synovial fluid between 10% and 40%, depending on the shear stress.

### Syringe-Filter Test

To assess whether the *in situ* formation of hydrogels slowed transport of the cationic nanoparticles across semipermeable membranes relative to similar nanoparticles that did not form the hydrogels, *in vitro* diffusion and forced convection tests were performed. Gels were formed by mixing cationic nanoparticles with human OA synovial fluid, and convective transport of the nanoparticles through a membrane was measured using a syringe filter with a 1-μm filter size, as described in the Materials and Methods section.

**Fig. 6:**
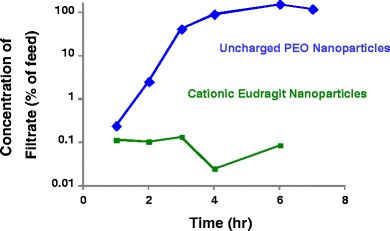
Forced convective transport of fluorescent nanoparticles in human OA synovial fluid through a 1-μm syringe filter: cationic 71:24:5 D10P:Eudragit RL100:MEH-PPV nanoparticles and uncharged 74:21:5:ethyl cellulose:PCL-PEO:MEH-PPV nanoparticles (fluorescence was measured at 580 nm).

Figure 6 shows a plot of the fluorescence at 580 nm of the filtrate in the syringe-filter test for two types of fluorescent nanoparticles in human OA synovial fluid: (1) cationic 71:24:5 D10P:Eudragit RL100:MEH-PPV nanoparticles and (2) uncharged 74:21:5:ethyl cellulose:PCL-PEO:MEH-PPV nanoparticles. As in other tests, the cationic nanoparticles formed a hydrogel upon mixing, whereas the neutral nanoparticles did not.

The uncharged PCL-PEO nanoparticles quickly passed through the filter, with the concentration of fluorescence in the filtrate increasing over the first several hours until it was equal to the starting concentration in the donor medium at the 4-hour timepoint. In contrast, few of the cationic Eudragit nanoparticles were forced through the filter by convective flow. At 7 h, the concentration of cationic nanoparticles in the filtrate was <0.1% of the concentration in the donor medium.

### Membrane Dissolution Test

Since both convective and diffusive transport are likely to influence nanoparticle clearance from the joint *in vivo*, the ability of the ionically cross-linked hydrogel to slow diffusive transport through a semipermeable synovial membrane was also measured *in vitro*.

**Fig. 7:**
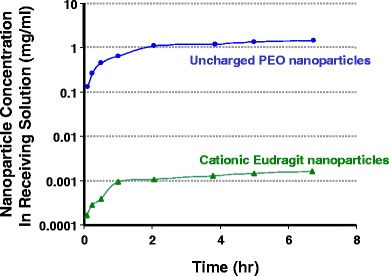
Diffusive transport of fluorescent nanoparticles in human OA synovial fluid through a semiporous 1-μm membrane: cationic 71:24:5 D10P:Eudragit RL100:MEH-PPV nanoparticles and uncharged 74:21:5:ethyl cellulose:PCL-PEO:MEH-PPV nanoparticles.

Diffusive transport of nanoparticles in human OA synovial fluid was measured *in vitro* using a semipermeable polypropylene membrane with a 1-μm pore size, as described in the Materials and Methods section. Figure 7 shows the percentage of nanoparticles transported through the membrane as a function of time for fluorescent nanoparticles similar to those tested in the syringe-filter test: cationic 71:24:5 D10P:Eudragit RL100:MEH-PPV nanoparticles and uncharged 74:21:5:ethyl cellulose:PCL-PEO:MEH-PPV nanoparticles. Again, transport was determined by fluorescence of the receptor solution at 580 nm. As shown in Fig. 7, the transport of the uncharged PCL-PEO nanoparticles was three orders of magnitude higher than for the cationic Eudragit nanoparticles over the course of the 7-h experiment. The concentration of the uncharged nanoparticles essentially equilibrated between the donor and receptor media within 2 h, whereas less than 0.1% of the charged nanoparticles passed through the membrane during the 7-h test.

### Hydrolysis of Peptide from Polymer

**Fig. 8:**
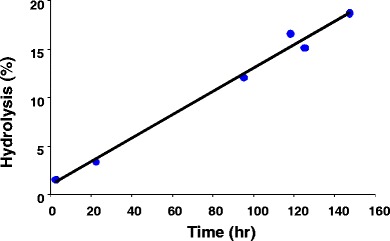
Hydrolysis of RFK peptide from D10PS-RFK-FITC nanoparticles over time.

An *in vitro* release study was performed to assess whether acceptable *in vivo* release rates of small molecules are likely to be achieved from the nanoparticles using hydrolyzable ester bonds. Using an ester linkage, a short fluorescent peptide consisting of three amino acids (i.e., RFK) was conjugated to a hydrophobically derivatized dextran polymer typical of those used in the core of the cationic nanoparticles. The hydrolytic release of the peptide was measured *in vitro* under physiological pH and temperature to assess the likely release rate *in vivo*. Figure 7 shows the *in vitro* release of the peptide from the D10PS-RFK-FITC polymer via ester hydrolysis at 37°C and pH 7.4. As shown in Fig. 8, approximately 20% of the RFK peptide was released over 147 h (~6 days), with a roughly linear release rate, demonstrating that the nanoparticles can release a small molecule at a sustained rate over an extended period.

### *In Vivo* Retention in Rats

**Fig. 9:**
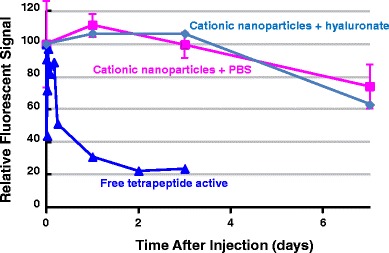
*In vivo* pharmacokinetics of 37.5:37.5:25 D10P:D20P-VivoTag:Eudragit RL100 nanoparticles versus free tetrapeptide in PBS

The retention of fluorescent 37.5:37.5:25 D10P:D20P-VivoTag:Eudragit RL100 nanoparticles within the knee joint after intra-articular injection in rats was measured using FMT. The animals did not show any adverse effects, such as signs of pain or inflammation, after intra-articular injection. Figure 9 summarizes the retention data for 37.5:37.5:25 D10P:D20P-VivoTag:Eudragit RL100 nanoparticles *versus* a fluorescently tagged free tetrapeptide. When the fluorescently labeled free tetrapeptide was injected intra-articularly, the fluorescence levels decreased to 23% of the initial concentration within 2 days. In contrast, 74% of the fluorescent tag incorporated into the nanoparticles via an ester bond remained in the knee 7 days after injection.

To determine if the endogenous level of hyaluronate in the knee joint was sufficient to form ionically cross-linked structures with the nanoparticles, a separate group of rats (*n* = 4) was given an intra-articular injection of exogenous hyaluronic acid immediately following the nanoparticle injection. The dynamic profile of fluorescence signal changes in the knees of these animals was very similar to those of the animals that did not receive the exogenous hyaluronate (Fig. 8). This suggests that the endogenous level of hyaluronate is adequate to bind the entire dose of nanoparticles ionically.

## Discussion

The goal of this study was to demonstrate the feasibility of using active compounds covalently attached to cationic nanoparticles to extend intra-articular retention time and prolong release of active small molecules.

In these studies, several nanoparticle formulations were made and characterized using *in vitro* methods. The nanoparticles were generally composed of a cationic polymer and at least one uncharged polymer. The nanoparticles described here were made by either an emulsion/evaporation method or by precipitation from solvent/nonsolvent mixing. They are believed to form core/shell structures in which the charged polymer predominantly resides on the surface of the nanoparticle and the neutral polymer(s) preferentially reside inside the core of the nanoparticle. This arrangement is plausible based on the more hydrophilic nature of the charged polymer relative to that of the uncharged polymers. In addition, the interaction observed between the nanoparticles and the anionic hyaluronate suggests a significant fraction of the cationic charge lies on the surface of the nanoparticle. Finally, the core/shell structure is consistent with x-ray photoelectron spectroscopy (XPS) surface analysis performed by the authors on dried nanoparticles having a composition similar to those described in these studies.

The results from these studies show that discontinuous, filamentous structures are formed when nanoparticles based on Eudragit RL100 or cationically modified dextran are added to whole human OA synovial fluid. The structures are believed to be ionically cross-linked materials consisting of cationic nanoparticles and anionic hyaluronate. Based on their appearance and on the water-swollen nature of hyaluronate, the filaments likely comprise a hydrogel, which forms a separate (discontinuous) phase in the synovial fluid (continuous phase). At sufficiently high concentrations of hyaluronate and cationic nanoparticles, the materials might form a single hydrogel phase.

Given the substantial structural differences between Eudragit RL100 and the cationic dextran tested, there is likely great latitude in the choice of cationic polymers. Modified dextrans are likely preferable to Eudragit RL100, since the substituted dextrans are biodegradable and form water-soluble products (e.g., dextran and propionic acid from dextran propionate), which can be cleared from the body through the kidneys. In addition, initial unpublished data of the authors suggests that intravenous injection of nanoparticles composed of uncharged ester-modified dextrans are well tolerated by rodents, although select anionic nanoparticles appear to be less so when administered systemically. More work is necessary to assess the safety of various derivatized dextrans when administered by different routes.

Like the cationic polymer, the uncharged core polymer can be varied while enabling effective gel formation by the cationic surface polymer. Different core polymers—D10P and ethyl cellulose—were used in the *in vitro* syringe-filter and membrane dissolution tests, respectively, and both resulted in the formation of the ionic gel by the cationic surface polymer.

The results described above suggest that selection of the surface and core polymers can be tailored to maximize safety without adversely affecting an ionic association or cross-linking needed to achieve nanoparticle retention within the targeted tissue.

The rheological testing results using human OA synovial fluid are promising, in that the viscosity of the synovial fluid was not drastically affected by the formation of the filamentous cross-linked hydrogel, being 40% more viscous at the lowest shear stress measured (0.1 Pa) and only 10% more viscous at higher shear stresses. In this testing, an attempt was made to use a nanoparticle suspension with a concentration and volume relative to the synovial fluid that is relevant for drug-delivery applications. Since the viscosity of the synovial fluid is mainly attributable to hyaluronate, it is encouraging that the viscosity of the synovial fluid did not increase much with addition of nanoparticles. This could be because the ionically associated material is not very strongly linked and/or that it forms a second, discontinuous phase in the synovial-fluid continuous phase and, thus, does not have a large impact on the rheological properties of the continuous phase. The result suggests that binding of a portion of the hyaluronate with the nanoparticles is not likely to adversely affect the important rheological functions of hyaluronate in the joint and, therefore, that the injection of a therapeutic agent using these cationic nanoparticles would not damage the knee joint by drastically changing the lubrication properties and other characteristics of the fluid in the joint. Likewise, the small size of the nanoparticles (~130 nm) and the diffuse, compliant nature of the cross-linked gel are not likely to cause cartilage damage or other mechanical damage in the joint.

The syringe-filter and membrane dissolution tests were used to measure convective and diffusional transport, respectively, of the charged nanoparticles, respectively, out of the ionically cross-linked hydrogel. Both types of transport are likely to be relevant for the elimination of therapeutic agents and their associated vehicles from the knee joints *in vivo*. In this study, the effect of the biomechanics of the knee—e.g., the pressure in the synovial cavity due to movement—on these two types of transport was not addressed. Further investigation of these effects will be pursued as part of future work.

The 1-μm pore size used in convective and diffusional transport tests was selected to approximate the size of the intercellular gaps reported for the synovial membrane surrounding the synovial cavity (31,32). This is likely an overestimate of the effective pore size, as macromolecular transport across the membrane suggests much smaller effective pore sizes—diameters of 66 to 118 nm for hyaluronidase (33) and 174 nm for dextran (34). From the optical microscopy images, the size of the gel filaments appeared to be many microns and, therefore, would not be expected to pass throught the 1-μm filter, although the ~100- to150-nm nanoparticles would be expected to pass through the filter if they were free in suspension and not incorporated into the gel.

The flow rate chosen for the *in vitro* convection studies (0.04 ml/cm^2^/hr) was approximately 20 times that reported for synovial fluid in the knee (35). It was chosen to determine if the gel could remain intact under the maximum anticipated physiological convective forces observed/measured in the joint. The 2-h time lag for transport of the neutral nanoparticles is due to limited mixing of the nanoparticle suspension with the synovial fluid that was prefilled into the syringe filter and expelled during the collection of aliquots at the initial timepoints. The higher concentration of fluorescence in the receptor solution relative to the donor suspension (150% of the initial concentration in the donor suspension) is likely due to incomplete mixing of the nanoparticles in the synovial fluid, resulting in inhomogeneity in the donor suspension. Some inhomogeneity was likely present because the viscous donor suspension was only gently mixed prior to the test to avoid shear-induced cleavage of the hyaluronate that might result from more vigorous mixing.

Dextran was chosen as the polymeric platform for this set of studies because of its long safety record as a blood plasma expander. Dextrans of up to 70 kDa are routinely used for that application and are degraded and cleared from the body safely. The dextran was derivatized as the propionate ester to render it insoluble for the production of nanoparticles while maintaining the biodegradability of the polymer through hydrolysis and esterase pathways. The esterification of the dextran with functional substituents has proven to be straightforward, which allows the polymer properties to be adjusted for optimum performance and safety.

The rate of release of a short peptide conjugated to the nanoparticles via ester hydrolysis was approximately 20% over 6 days, which is appropriate for providing approximately 1-month duration of exposure of a therapeutic agent after injection into the knee cavity, assuming a similar rate *in vivo*. The ester hydrolysis rate measured was similar to rates for similar esters reported in the literature. For example, Larsen reported half lives of between 280 and 312 h in human OA synovial fluid for a naproxen prodrug formed from dextran backbones (36). Ester hydrolysis of a succinate group linked to the n-terminus is ideal for this particular peptide, and this approach is likely applicable to a wide range of active molecules. For nonpeptide small active compounds, alternate conjugation chemistries can be tailored to the chemical structure to achieve the desired release profile. For large molecules, including proteins, noncovalent incorporation into nanoparticles can be used, for example by using polyionic interactions between the protein and the synthetic polymer used in the nanoparticle carrier.

The *in vivo* examination of the cationic nanoparticles demonstrated that exogenously injected hyaluronate was not necessary to increase the retention time. The result suggests that the concentration of endogenous hyaluronate in the synovial fluid is sufficient for association to the nanoparticles and for slowing their otherwise rapid transport out of the synovium.

The FMT signal is due to the ester-linked fluorophore. Therefore, in the current study, fluorescent signal in the knee is lost when either the nanoparticles themselves are transported out of the joint or when the fluorophore is released from the nanoparticles by hydrolysis and exits the joint, leaving the nanoparticles behind. The latter mechanism is preferred for sustained drug-delivery applications, so that the drug is released within the joint rather than after exiting the joint with the nanoparticles. Determining the extent to which the decrease in fluorescence in the knee is due to transport of hydrolyzed free fluorescent peptide *versus* transport of peptide in nanoparticles would require additional experiments that were beyond the scope of this study. However, because the loss of fluorescence in the knee *in vivo* closely matched the *in vitro* ester hydrolysis rate of approximately 20% per week, most of the signal decrease observed over the 1-week *in vivo* study was likely due to transport of fluorophore out of the joint after hydrolysis from the nanoparticles, rather than from transport of intact nanoparticles themselves out of the joint. This question will be addressed in future studies in which the nanoparticles and modeled drug will be labeled separately.

Future work will focus on testing the described nanoparticle formulations with known DMOADs (e.g., MMP-13 and aggrecanase small-molecule inhibitors) to determine the effectiveness of this approach in blocking cartilage degradation and bone erosion in various animal models of OA.

## Conclusions

The feasibility of using cationic nanoparticles to increase the retention time of drugs in the knee joint of rats has been demonstrated, offering a new promising approach for achieving increased retention and sustained delivery of drugs in the knee cavity for treatment of OA or other rheumatic diseases. This platform would enable less frequent injections, decreasing both cost and patient discomfort, while potentially increasing the effectiveness of treatments by providing a more stable drug concentration.

## Abbreviations

ACN: acetonitrile
D10P: dextran 10 propionate
D10PS: dextran 10 propionate succinate
D20AQA-TR: dextran 20 acetate quaternary amine-Texas Red
D20P: dextran 20 propionate
Dextran 10: dextran with a molecular weight of 10 kDa
Dextran 20: dextran with a molecular weight of 20 kDa
DLS: dynamic light scattering
DMOADs: disease-modifying osteoarthritis drugs
ELPs: elastin-like polypeptides
FITC: fluorescein isothiocyanate
FMT: fluorescence molecular tomography
HPLC: high-performance liquid chromatography
IACUC: institutional Animal Care and Use Committee
LAAS: laboratory animal anesthesia system
MEH-PPV: poly[2-methoxy-b-(2-ethylhexyloxy)-1,4-phenylenevinylene]
MeOH: methanol
NSAIDs: nonsteroidal anti-inflammatory drugs
OA: osteoarthritis
PBS: phosphate buffered saline
PCL-PEO: polycaprolactone-b-co-polyethyleneoxide
PES: polyethersulfone
PK: pharmacokinetic
PLA: poly(lactide)
PLGA: poly(lactide-co-glycolide)
PLLA: poly-L-lactide acid
RFK-FITC: RFK peptide labeled with fluorescein isothiocyanate
TFA: tetrafluoroacetic acid
THF: tetrahydrofuran
UV: ultraviolet
UV–vis: ultraviolet visible
XPS: X-ray photoelectron spectroscopy
